# Identification of reference genes for RT-qPCR data normalization in Gammarus fossarum (Crustacea Amphipoda)

**DOI:** 10.1038/s41598-018-33561-1

**Published:** 2018-10-15

**Authors:** Kahina Mehennaoui, Sylvain Legay, Tommaso Serchi, François Guérold, Laure Giamberini, Arno C. Gutleb, Sébastien Cambier

**Affiliations:** 1 Environmental Research and Innovation (ERIN) Department, Luxembourg Institute of Science and Technology (LIST), 5, avenue des Hauts-Fourneaux, Esch-sur-Alzette, Luxembourg; 2 Laboratoire Interdisciplinaire des Environnements Continentaux (LIEC), CNRS UMR 7360 Université de Lorraine – Metz, France

## Abstract

Gene expression profiling via RT-qPCR is a robust technique increasingly used in ecotoxicology. Determination and validation of optimal reference genes is a requirement for initiating RT-qPCR experiments. To our best knowledge, this study is the first attempt of identifying a set of reference genes for the freshwater crustacean *Gammarus fossarum*. Six candidate genes (*Actin, TUB, UB, SDH, Clathrin* and *GAPDH*) were tested in order to determine the most stable ones in different stress conditions and to increase the robustness of RT-qPCR data. *SDH* and *Clathrin* appeared as the most stable ones. A validation was performed using *G. fossarum* samples exposed for 15 days to AgNO_3_, silver nanoparticles (AgNPs) 40 nm and gold nanoparticles (AuNPs) 40 nm. Effects on *HSP90* were evaluated and data normalized using *Clathrin* and *SDH*. A down-regulation of *HSP90* was observed when *G. fossarum* were exposed to AuNPs 40 nm whereas no effects were observed when *G. fossarum* were exposed to AgNPs 40 nm. This study highlights the importance of the preliminary determination of suitable reference genes for RT-qPCR experiments. Additionally, this study allowed, for the first time, the determination of a set of valuable genes that can be used in other RT-qPCR studies using *G. fossarum* as model organism.

## Introduction

Understanding the mechanisms underlying the effects of stressors on organisms needs sensitive analytical techniques that can cover and link responses observed at different biological levels (from molecular to individual responses). One of the most reliable techniques is the measurement of changes or alterations in gene expression in response to an external stimulus^1^. Recent advances in “omics” and bioinformatics methodologies applied in ecotoxicological studies provided a new angle of studying non-model organisms, opening new ways in determining new molecular biomarkers (genes) as an alteration of their regulation may influence the fitness of organisms^2,3^. Reverse transcription quantitative polymerase chain reaction (RT-qPCR) is currently described as one of the most reliable techniques to assess these changes due to its effectiveness, sensitivity and reproducibility^1,2,4^. This method, which allows studying the expression of a set of selected genes in an organism, requires multiple critical quality controls in order to obtain robust results. This includes RNA purity and integrity control, genomic DNA contamination assessment, evaluation of PCR primer efficiency and specificity and, in case of relative quantification of gene expression, the identification of suitable reference genes for data normalization^1,5,6^.

Reference genes are described to be stable regardless of the exposure conditions and overall treatments groups within an experimental design, making them suitable for data normalization of genes of interest. Therefore, their determination is crucial for a good analysis of RT-qPCR results^2,5,7,8^. Furthermore, combination of a set of the most stable reference genes is fundamental to avoid misinterpretation of the results that may occur when using a unique reference gene^1^. Consequently, a preliminary experimental determination of the optimal reference genes is necessary before each new experimental design^5,8^. For this purpose, many software programs and tools have been developed in order to determine the best reference genes. This selection is performed following a ranking method based on their stability across different stress conditions^5,7,9^. Unfortunately, many ecotoxicological studies are still using only one, or a set of reference genes selected from previous studies, applying different experimental designs, making them sometimes improper for data normalization. Classically known housekeeping genes such as *Actin, Tubulin* (*TUB*), *glyceraldehyde-3-phosphate dehydrogenase* (*GAPDH*), and 18 *S ribosomal RNA* are used as reference genes^2,4,6,8,10^. However, recent studies demonstrated that some of these genes might not be suitable as they are not always stable depending on the experimental design, studied stress and organisms leading sometimes to the misinterpretation of the results^8,11^.

Amphipods of the genus *Gammarus*, and more specifically *Gammarus fossarum*, represent a major part of the aquatic macroinvertebrates assemblage^12,13^. They are used in several ecotoxicological studies for their high ecological relevance linked to their wide distribution all over Europe and their major functional role in litter breakdown process and nutrient cycling^14,15^. Their well-documented sensitivity to different kinds of pollutants make them good model organisms for ecotoxicological studies^16–20^. However, to our knowledge, few studies investigated their responses to exogenous stress at the molecular level, such as the vitellogenin expression following an estrogenic stress^21^, the antioxidant responses via *catalase* and *MnSOD* gene expression following exposure to gold nanoparticles (AuNPs)^22^ and the identification of proteins expression profiles during spermatogenesis^21–23^, and the expression of the *NaKATPase* following exposure to cadmium^24^. Nevertheless, there are still no reference genes properly characterized and dedicated to the amphipod *G. fossarum* although few studies tested a limited number of genes on *G. fossarum* without any algorithms, including *β-actin*^22^, *Elongation factor-1 alpha* (*eiF-1a*)^10,24^, 18 *S and GAPDH*^10^. Reference genes are mandatory for an accurate data normalization of gene expression^1^. Therefore, the aim of the present study is to fill this gap and to determine a suitable tool kit made of a set of reference genes for data normalization of RT-qPCR experiments using *G. fossarum*. Some studies are available on the identification of reference genes on other aquatic organisms such as the clam *Ruditapes philippinarum*^8^, flatworms *Macrostomum ligano and Schmidtea mediterranea*^25^, green algae *Ulva linza*^26^, *Daphnia magna*^6^ etc. These studies highlighted the importance of an accurate selection of reference genes and allowed the accurate selection of six candidate reference genes for the present work^4,8,9^. The reliability of the selected genes for RT-qPCR were tested in different exposure conditions using *G. fossarum* exposed to AgNO_3_, silver nanoparticles (AgNPs) and gold nanoparticles (AuNPs).

## Results

### Stability of the candidate reference genes in *G. fossarum*

To identify the most appropriate set of reference genes for *G. fossarum*, eight candidates were tested. The genes included some frequently used ones (*Actin, TUB, eiF-1a*, 18 *S and GAPDH*) and less common ones, namely, succinate dehydrogenase (*SDH*), ubiquitin (*UB*) and *Clathrin*. Stability coefficients were determined using five different methods, namely, GeNormPlus^27^, NormFinder^28^, BestKeeper^7^, RefFinder^29^ and the comparative delta-CT method^30^. Stability coefficients were compared in order to determine the most stable gene sub-set. The ranking was performed only on amplified genes (*Actin, TUB, GAPDH, SDH, UB* and *Clathrin;* Figure S3).

The ranking of the studied genes varied dependently on the algorithms used (Table 1). Interestingly, the same ranking was obtained using the most commonly used softwares, GenormPlus and NormFinder, with *Clathrin* and *SDH* as the most stable genes and *UB* as the least stable one. In the same way, RefFinder and the comparative delta-CT method identified *Clathrin* and *SDH* as the most stable genes. *GAPDH* was the third most stable gene according to GenormPlus, NormFinder and RefFinder, whereas, the rankings were different for the least stable ones (Table 1) as *Actin* was assigned the highest score by the comparative delta-CT method and RefFinder (Table 1).

**Table 1:** Ranking of candidate reference genes according to the five algorithms used.

Ranking	GeNorm		NormFinder		BestKeeper		Comparative delta Ct		RefFinder	
	Gene	Stability coeff	Gene	Stability coeff	Gene	Stability coeff	Gene	Stability coeff	Gene	Stability coeff
1	*Clathrin*	0.433	*Clathrin*	0.105	*Clathrin*	0.670	*Clathrin*	1.035	*Clathrin*	1.000
2	*SDH*	0.470	*SDH*	0.198	*Actin*	0.624	*SDH*	1.067	*SDH*	1.682
3	*GAPDH*	0.550	*GAPDH*	0.265	*SDH*	0.571	*TUB*	1.188	*GAPDH*	3.464
4	*Actin*	0.593	*Actin*	0.292	*UB*	0.575	*GAPDH*	1.190	*TUB*	3.464
5	*TUB*	0.652	*TUB*	0.465	*GAPDH*	0.459	*UB*	1.232	*UB*	5.000
6	*UB*	0.703	*UB*	0.520	*TUB*	0.395	*Actin*	3.217	*Actin*	6.000

As BestKeeper’s ranking is based on correlation factor (r; the closer to 1 the better) and standard deviation (the bigger, the worse)^7,31^, the obtained results differed from the other algorithms as *Clathrin* and *Actin* appeared as the most stable genes whereas *GAPDH* and *TUB* were the least stable ones. According to BestKeeper, *SDH* was the third most stable gene (Table 1).

**Figure 1:**
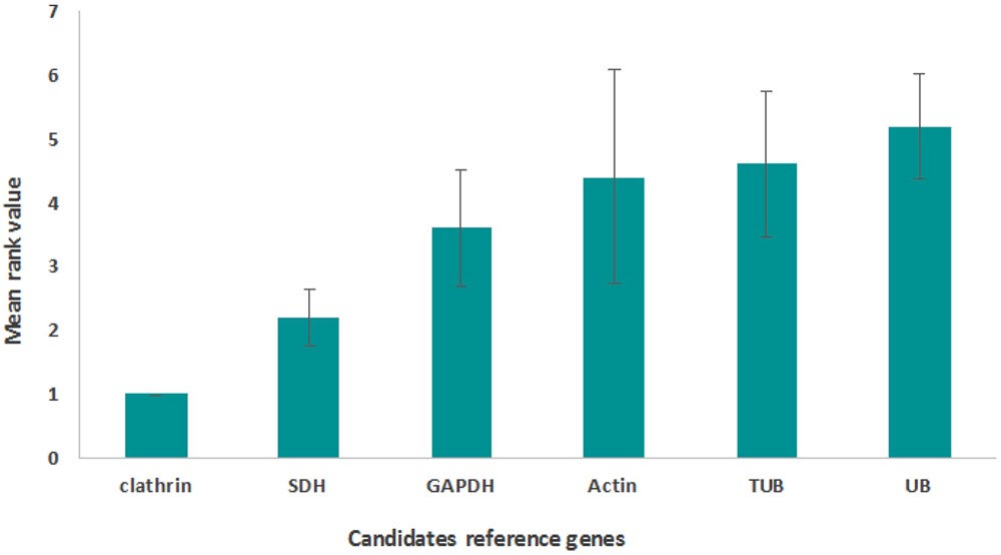
Global ranking of candidate reference genes in *G. fossarum*. A number (from 1 to 6) was assigned to each stability coefficient. A mean rank was generated and error bars represent standard deviations.

In order to determine the stability of each gene, a global ranking was generated by assigning a number (from 1 to 6 where 1 is the most stable gene) to each stability coefficient presented in Table 1 and by averaging them^1,9^. This allowed the confirmation of the high stability of *Clathrin* and *SDH* (Fig. 1). *Ubiquitin* appeared as the least stable gene followed by *TUB* and *Actin* (Fig. 1).

### Optimal number of reference genes for data normalization in *G. fossarum* using GeNorm

In order to calculate the optimal number of reference genes for data normalization in *G. fossarum*, GeNormPlus was used to generate the pairwise variation (Vn/Vn_+1_) between two normalization factors (NF/NF_n+1_). Indeed, only GenormPlus allows an estimation of the optimal number of reference genes to use in a specific experimental design.

**Figure 2:**
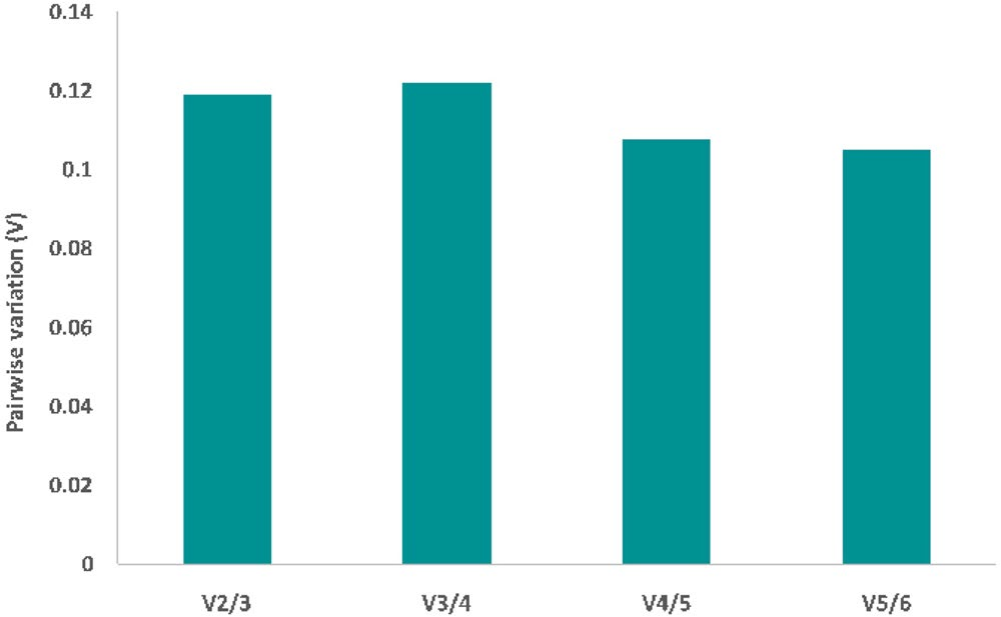
Determination of the optimal number of reference gens for data normalization in *G. fossarum* exposed to AgNO_3_, AgNPs 40 nm and AuNPs 40 nm. The pairwise variation (Vn/Vn_+1_) was calculated between normalization factors NF/NFn_+1_. The recommended cut-off threshold of 0.15 was applied in this study.

The analysis conducted on 31 samples of *G. fossarum* exposed to AgNO_3_, AgNPs 40 nm and AuNPs 40 nm, showed that the optimal number of reference genes is 2 as the V value is below the cut-off threshold of 0.15 making the addition of a third gene unnecessary (Fig. 2). As previously described, GeNormPlus identified *Clathrin* and *SDH* as the best combination for data normalization. In the same way, NormFinder determined *Clathrin/SDH* as the best pair as these two genes showed the highest stability coefficient (Table 1).

### Validation of the selected reference genes for *G. fossarum*

**Figure 3:**
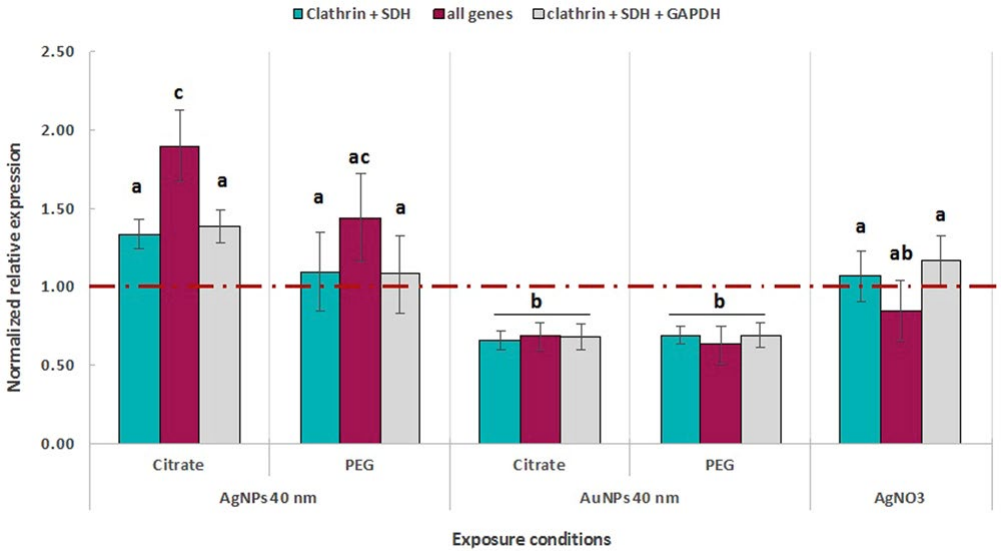
*HSP90* expression analysis using different normalization strategies. Error bars indicate the standard errors of the means (n = 4). Different letters (**a**–**c**) indicate significant differences at *P* < 0.05. Red line represents the relative expression to the control groups that is set at 1.00.

In order to validate *G. fossarum* reference genes, the expression of a general stress-related gene, the heat shock protein 90 (*HSP90*), was evaluated. Expression profiles of *HSP90* were studied on *G. fossarum* exposed for 15 days to 0.5 μg. L^−1^ of AgNO_3_, CIT-AgNPs 40 nm, PEG-AgNPs 40 nm, CIT-AuNPs 40 nm and PEG-AuNPs 40 nm. Data were analysed using the Biogazelle qbase + software and normalized using *Clathrin* and *SDH*. Additionally, non-normalized data were analysed (Fig. S1). As shown in Figs 3 and S1, a significant decrease in *HSP90* expression was observed when *G. fossarum* were exposed to 0.5 μg. L^−1^ of CIT-AuNPs 40 nm and PEG-AuNPs 40 nm (One-way ANOVA, *P* < 0.001) while none of the tested AgNPs or AgNO_3_ impacted *HSP90* expression (Fig. 3, One-way ANOVA, *P* > 0.05). However, when data were normalized using all the six reference genes, the statistical analysis showed a significant induction of the expression of *HSP90* after treatment with CIT-AgNPs 40 nm (Fig. 3, One-way ANOVA, *P* < 0.05). Data were also normalized using the three best reference genes *Clathrin, SDH* and *GAPDH*, but results do not differ by adding this extra reference genes. (Fig. 3, One-way ANOVA, *P* > 0.05) enhancing that adding a third gene for data normalization was unnecessary for our experimental design (Fig. 3).

## Discussion

Despite the rapid advances in the “omics” field marked by the development of next generation sequencing (NGS) methods, RT-qPCR remains one of the most accurate and reliable techniques for targeted gene expression and NGS data validation. This method is recognized for its capacity to highlight sensitive changes in gene transcription levels^1,25^. However, for an accurate interpretation of RT-qPCR results, in terms of relative gene expression, one of the most important criteria is the selection of suitable reference genes^32^, which, to the best of our knowledge, are lacking for *G. fossarum* species. Previous studies have already investigated molecular responses in *Gammarus* sp., exposed to AuNPs, temperature and ammonia, using *Actin*, or *GAPDH* as reference genes^22,33^ on the assumption that these genes were stable and without prior experimental verifications. Such approaches might lead to misinterpretation of RT-qPCR results^5,26,34^ since the expression of these reference genes might be influenced by biotic or abiotic stress as well as developmental stage and tissue type^4,8,35^. It is well established that a single reference gene could not be applied to all experimental designs^1,5^. Therefore, a case-specific choice of the best reference genes for RT-qPCR is mandatory. In the present study, eight candidate genes, which were selected on the basis of their use as reference genes in other organisms, have been tested in adult *G. fossarum* males, regarding a contamination with AgNO_3_, AgNPs and AuNPs, for their appropriateness to be used as reference genes in transcriptomic studies^4^. The efficiency and specificity of the designed primers have been checked. Six of the eight tested couple of primers showed correct PCR efficiency and specificity during the PCR ensuring robust and precise results interpretation^5^, and confirmed that our RT-qPCR experiments comply with the known recommendations^4,5^. Within the set of genes tested, *eiF-1a* and 18S could not be amplified in the present qPCR experimental conditions regardless of the primers tested and were therefore excluded from the analyses. Five different algorithms were used to determine optimal reference genes to normalize our data. The global ranking allowed us to identify *Clathrin* and *SDH* as the most stable genes. These results are in accordance with previous studies that described *SDH* as a suitable reference gene in *Rhodnius prolixus*^11^ and in red abalone *Haliotis rufescens*’ gonad and digestive tissues^36^. *Clathrin* was also identified as one of the most stable genes in *Lilium formolongi*^37^.

It is important to notice that our ranking of the candidate reference genes shows that the most commonly used genes in transcriptomics studies are the least stable ones in this study. This observation is in agreement with other recent studies which showed that *GAPDH, 18s rRNA*, *Actin* and *TUB* were not good candidates as reference genes^4,8,25,38^. *GAPDH*, commonly considered as a housekeeping gene, plays an important role in energetic metabolism and its expression was described as significantly impacted in bivalves, like *Mytilus* spp. or *Crassostrea gigas*, exposed to different environmental stress (e.g. harbor pollution), and in *Haliotis discus hannai* under heavy metal stresses conditions^4,38^. On the contrary, other studies showed that *GAPDH* was one of the most stable genes in two flatworm species, *Macrostomum lignano* and *Schmidtea mediterranea*, exposed to cadmium^25^ and in *R. prolixus* in different physiological conditions and feeding states^11^. These observations indicate that *GAPDH* should not be considered as suitable for data normalization without prior validation. Other studies demonstrated that selection of unstable genes like *Actin* and *18S rRNA* as reference genes greatly altered interpretation of data for *Cathepsin D* in primary *Ruditapes philippinarum* hemocytes exposed to copper^8^ and for *Catalase* and *NADPH-dependent thioredoxin reductase A (NTRA)* gene expression in *Arabidopsis thaliana* exposed to Zn and Cd, respectively^32^. Similar observations were done for *TUB* and *UB* in *Haliotis discus hannai*^4^. These results are in agreement with our observations in *G. fossarum* for which classical reference genes like *Actin, TUB, UB* and *GAPDH* appeared among the least stable genes. The present study shows that it is crucial to experimentally assess the stability of reference genes for each species in each experimental design applied before they can be selected as housekeeping genes^8^.

In addition, the stability of the present reference genes was verified by exposing *G. fossarum* to AgNO_3_, and differently coated (CIT and PEG) AgNPs 40 nm and AuNPs 40 nm for 15 days and studying the expression of *HSP90*. The gene *HSP90* is involved in the regulation of proteostasis under both physiological and stress conditions. It is involved in protein folding as a chaperone, DNA repair and immune responses^39^. Together with *HSP70, HSP90* is also known to play a role in keeping heat shock factor 1 (*HSF1*) inactive, which induces the expression of *HSP90* in stress conditions^39,40^. Therefore, it was used as a target gene for general stress indication in the present study. As the aim of the present study is also to define the influence of the accurate selection of reference genes on the expression of target genes, data were normalized using, firstly *Clathrin* and *SDH*, which were indicated by GeNormPlus to be the most stable genes, then, *Clathrin, SDH* and *GAPDH* and finally with the whole set of six candidate reference genes. Moreover, the use of normalized and non-normalized data in the relative expression analysis of *HSP90* was compared. This approach allowed to check qPCR data accuracy as the real *in vivo* gene expression are suggested to be situated between normalized and non-normalized data^32^. Moreover the relative expression of *HSP90* of *G. fossarum* exposed to the chemical relatively to the control from the non-normalized data and the normalized data revealed the same pattern still with less variability when the data are normalized. When *Clathrin* and *SDH* are used for normalization, a significant decrease in *HSP90* expression was observed in *G. fossarum* exposed to CIT-AuNPs 40 nm and PEG-AuNPs 40 nm. However, when data were normalized using *Clathrin, SDH* and *GAPDH*, results do not differ when adding *GAPDH* as an extra reference genes making the addition of a third gene unnecessary which fits to the recommendation of the MIQE guidelines (M value < 1 and V value < 0.15)^32^. Furthermore, data normalization using all the six studied reference genes, led to the detection of an upregulation of *HSP90* in *G. fossarum* exposed to CIT-AgNPs 40 nm, which in this case is to be considered as an overestimation of the response as “incorrect references” were used. This is in accordance with what has been previously reported^25^. Normalization of *HSP90* expression in *M. lignano* exposed to Cd using all the nine reference genes tested led to a high variability between replicates. Authors stated that this observation could be linked to an important variability between replicates that lower the resolution of detecting differences between their different conditions^25^. However, when data were normalized using the three most stable genes, no significant differences in *HSP90* expression were observed^25^. Another study showed that a non-optimal selection of the best combination of reference genes may lead to statistical misinterpretation^11^. Authors showed that data normalization using an unstable gene such *eiF-1a* in *R. prolixus* led to a clear but false increase in an olfactory gene, *RproIR76b* expression while no statistical differences were observed when data were normalized using the most stable genes^11^. Moreover, another study underlined the importance of selection of the appropriate reference genes with the highest stability coefficient as data normalization of metallothionein expression in abalone exposed to copper led to an underestimation or overestimation of the effects when data were normalized using unstable reference genes^4^. These results highlight once more the importance of an experimental validation of reference genes in addition to the selection of the optimal number and the appropriate genes for data normalization^5,11,25^.

## Conclusions

This study provides, for the first time, a set of reference genes suitable for normalization of RT-qPCR data obtained from *G. fossarum* samples. Eight candidate genes were identified and tested. Five different algorithms allowed the identification of the most stable subset of genes. *Clathrin* and *SDH* were identified as the most stable genes in our applied experimental design, while widely used reference genes were unsuitable in *G. fossarum* in the present work, with *eiF-1a and 18S* being non-amplified. Our results highlight how important and crucial it is to experimentally define and validate a subset of reference genes for each RT-qPCR experiment. Moreover, the identified set of reference genes represent a solid tool kit for further targeted gene expression experiments using *G*. *fossarum* as a model organism. Although the present study allowed the identification of a first set of reference genes in *G*. *fossarum* opening up the way for future studies using this species as a model organism, further investigation are still needed in order to strengthen the present genes-set in order to fits the MIQE guideline by testing at least 10 candidate reference genes.

## Materials and Methods

### Organisms sampling and acclimation

*G. fossarum* were collected at an unpolluted stream (49°48′24.9″ N and 06°04′53.2″ E, Schwaarzbaach, Colmar-berg, Luxembourg)^19,20,41^. Animals were collected using a hand net and were sorted in the field. They were immediately brought to the laboratory in river water, where they were kept at 12 °C. In order to avoid influence of gender on the studied parameters, only adult males were kept for the experiment^17,42^. They were selected from precopula pairs or based on sexual dimorphism like gnatopode size^19^. Adult males were then acclimated to laboratory conditions^19,43^. The acclimation was conducted in two steps. First, Gammarids were acclimated for 72 h to mineral water (Volvic, France) by progressively changing field water to Volvic water (30% v/v, 50% v/v, 100% v/v) Then, a stalling period of 10 days was conducted in 100% Volvic water^19,20,43^. The acclimation was performed under controlled conditions at 12 °C with a 16 h light/8 h dark photoperiod. Volvic water was aerated and changed every 24 h to avoid organic matter accumulation and potential increase of ammonium, nitrite and nitrate. During the acclimation period, Gammarids were fed ad libitum with alder leaves (*Alnus glutinosa*).

### AgNO_3_, AgNPs and AuNPs contamination

At the end of the acclimation period, in addition to control group for each experimental condition, animals were exposed to 0.5 μg.L^−1^ of AgNO_3_, AgNPs 40 nm and AuNPs 40 nm, either stabilized with citrate (CIT-AgNPs 40 nm and CIT-AuNPs) or coated with polyethylene-glycol (PEG-AgNPs 40 nm and PEG-AuNPs 40 nm), for 15 days at 12 °C with a photoperiod of 16 h light and 8 h darkness^19^. Exposure medium (Volvic water) was changed every 72 h. Food (*Alnus glutinosa* disk leaves) was added every 48 h. At the end of the exposure period, a pool of four Gammarids per replicate (n = 4) were gently dried, flash-frozen in liquid nitrogen and stored at −80 °C in RLT buffer (Cat ID: 79216, Qiagen, Leusden, The Netherlands) supplemented with 1% β-mercaptoethanol until RNA extraction.

### Gene identification and qPCR primer design

#### Gene identification

In order to identify and amplify putative genes from *G. fossarum*, an initial data mining was performed on the raw reads previously sequenced by (Trapp *et al*.)^23^. Eight genes were selected in order to be tested as reference genes (*Actin, Tubulin, 18S, eiF-1a, Clathrin, GAPDH, SDH, UB*). Reads were mapped to the sequences of *Hyalella azteca* (https://www.hgsc.bcm.edu/arthropods/hyalella-azteca-genome-project), a closely related species for which the transcriptome is available, with the following criteria: the mapped reads must have multiple hits lower than 10, a minimum of 80% identity and 80% coverage with the reference. Mismatch costs was set at 2 (medium) and deletion/insertion cost at 3 (highest stringency). A consensus sequence was generated from the mapped reads for each gene (Table 2).

**Table 2:** Identification of *Gammarus fossarum* gene sequences (Sequence provided in Supplementary Material in Table S1). *GAPDH*: glyceraldehyde-3-phosphate dehydrogenase, *SDH*: succinate dehydrogenase, *HSP90*: heat-shock protein 90.

Genes	*Hyalella azteca*	*G. fossarum* consensus sequences	NCBI Blastx		
	Accession number	Accession number	Homology	Identification	Accession number
*Actin*	XM_018157137.1	MF940257	97%	Actin, partial [*Hoplolaimus galeatus*]	AEM45650.1
*Tubulin*	XM_018153872.1	MF940258	96%	PREDICTED: Tubulin alpha-8 chain-like isoform X1 [*Serinus canaria*]	XP_009098159.2
*Ubiquitin*	XM_018170409.1	MF940259	96%	ubiquitin conjugating enzyme-3 [*Eriocheir sinensis*]	ADF45343.1
*GAPDH*	XM_018154227.1	MF940254	95%	Putative glyceraldehyde-3-phosphate dehydrogenase [*Gammarus locusta*]	CAQ60115.1
*SDH*	XM_018156499.1	MF940255	96%	PREDICTED: succinate dehydrogenase [ubiquinone] iron-sulfur subunit, mitochondrial-like [*Hyalella azteca*]	XP_018011988.1
*Clathrin*	XM_018171236.1	Sequence provided in Table S1	93%	PREDICTED: Clathrin light chain-like isoform X1 [*Hyalella azteca*]	XP_018025977.1
*HSP90*	XM_018155941.1	MF04256	98%	PREDICTED: heat shock protein HSP 90-alpha-like [*Hyalella azteca*]	XP_018022683.1

In order to verify whether the obtained sequences are coding for a protein, translations of the obtained nucleotide sequences were performed using ExPASy translation tool (http://web.expasy.org/translate/). Finally, a Blastx search was performed against non-redundant protein databases from the National Centre for Biotechnology (NCBI) to check the identity of the selected sequences. (Table 2). The putative genes identified were then sequenced using SANGER method in order to confirm the identity of the selected sequences (Table S1). Only 6 genes from the eight selected were amplified and identified (Table S1).

#### Primer design

All the primers were designed using Primer3Plus (http://primer3.ut.ee) with the following criteria: primer size between 18 and 25 base pairs, GC content between 40% and 60%, amplicon size from 80 to 150 base pairs, primer annealing temperatures in the 58–61 °C range. Primers were checked using NetPrimer (http://www.premierbiosoft.com/netprimer/) for secondary unexpected structures. PCR efficiency was evaluated using decreasing five-fold dilutions from cDNA pool (from 25 ng to 0.04 ng and no template control) and calculated based on the equation [10^(−1/slope)]−1^ (Fig. S2; Table S3). A melting curve was performed at the end of each run, in order to assess the specificity of the amplified products and those amplified products were sequenced as the proof that the correct genes were amplified (Table S1, Fig. S3). All tested genes displayed one clear peak and were therefore retained for analyses^20^ (Fig. S3). Primers sequences, amplicon size, and melting temperature are described in Table 3.

**Table 3:** List of primers of the candidate reference genes and target gene *HSP90*.

Name	Sequence (5′ → 3′)	Amplicon Length (bp)	Amplicon Tm (°C)	PCR Efficiency	Regression Coeff. (R^2^)
*Actin_F*	CTCATGCTATCCTTCGTCTTGA	103	78	2.02	0.999
*Actin_R*	CGTTCAGCGGTGGTTACAA				
*Tubulin_F*	CGGCTGTTGTTGAACCTTAC	93	81	2.09	0.999
*Tubulin_R*	AGATGGCCTCATTGTCAACC				
*GAPDH_F*	GTCCGTCTCGCTAAGGAGTG	94	85	1.91	0.999
*GAPDH_R*	TGTATCCGAGGTAGCCCTTG				
*SDH_F*	GGAAGAAGCTGGATGGTCTG	87	84	1.98	0.998
*SDH_R*	ACTTGTCTCCGTTCCACCAG				
*Ubiquitin_F*	CCCACGATACTCCCTTTGAA	82	79	2.01	0.991
*Ubiquitin_R*	ACAATCGGTGGCTTGTTAGG				
*Clathrin_F*	ATCGCCAAGCTTTGTGACTT	107	85	1.99	0.999
*Clathrin_R*	GCTTTGATAGGCGGACTCTG				
*HSP90_F*	CTGGTTTCTTCTCCCTGCTG	135	85	1.99	0.995
*HSP90_R*	GATCTCGAGGTGCTTCTTGG				

### RNA extraction, cDNA and RT-qPCR

*G. fossarum* tissues were ground on ice using a pellet pestle tissue grinder. Homogenates were centrifuged at 250 g for 5 min at 4 °C to remove cuticle fragments as previously described^19^. Total RNA was extracted using RNeasy mini kit (Qiagen, Leusden, The Netherlands) according to the manufacturer’s instructions for cells and animal tissues (including DNase treatment)^20,44,45^. RNA concentrations and purity were assessed measuring the absorbance at 230, 260 and 280 nm using Nanodrop ND-1000 (ThermoScientific, Villebon-sur-Yvette, France). Finally, RNA integrity was checked using the RNA Nano 6000 assay (Agilent Technologies, Diegem, Belgium) and 2100 Bioanalyzer (Agilent, Santa Clara, CA, USA)^44^. All RNA samples displayed no degradation patterns (sharp peaks and clean baseline).

1 µg of the extracted RNA were reverse-transcribed into cDNA using the Protoscript II reverse transcriptase (New England Biolabs, Leiden, The Netherlands) and random primers, according to the manufacturer’s instructions. PCR were performed using a 384-well plate design in 10 μL with final concentrations as follows: 1X MasterMix, 100 nM for each primer and 0.8 ng. µL^−1^ of cDNA. An automated liquid handling robot (epMotion 5073, Eppendorf, Hambourg, Germany) was used to properly prepare the 384-well plates. qPCR runs were performed using the Takyon Low ROX SYBR MasterMix dTTP Blue Kit (Eurogentec, Liège, Belgium) on a ViiA 7 Real-Time PCR System (Thermo-Fisher, Waltham, MA, USA) in a 10 µL final volume^46^. All reactions were performed in technical triplicates and repeated on four biological replicates. The PCR conditions consisted on an initial denaturation step at 95 °C for 3 min, followed by 40 cycles of denaturation at 95 °C for 15 sec and annealing/extension steps at 60 °C for 60 sec. The relative gene expression was calculated including PCR efficiency using Biogazelle qbase Plus software 2.5 with the ^ΔΔ^Cq method (Cq’s are provided in Table S2).

### Stability of the candidates’ reference genes

The stability of the selected genes was analysed following the MIQE guidelines (Table S4) using five different methodologies. GeNormplus performs a pairwise comparison and generates the M-value which consist of a comparison of the variation of a gene compared to all the remaining candidates^27^ while NormFinder calculates both a single best gene (best gene) and an optimal gene pair (best pair) as the best pair may compensate the expression in the different experimental groups^1,28^. BestKeeper is based on assigning the correlation factor of each gene with the geometric means of all genes^7,31^ while RefFinder is an online very easy to use platform which compiles the three most popular algorithms for reference gene validation based on an input of Cq values only^29,31^. This method assigns an appropriate weight to each gene and calculates the geometric means of their weight for the final ranking with the lower score indicating the most stable gene^47^. Additionally, a simple delta-Ct comparison approach was applied^30^. This approach consist on comparing relative expression of ‘pairs of genes’ within each sample, then on this basis the stability of each reference genes candidates was ranked regarding the repeatability of the gene expression among the samples.

### Statistical analyses

Normal distribution of dataset was checked using a Shapiro-wilk test. Homogeneity of variances was checked using a Levene test. A one-way ANOVA followed by a Fisher-LSD post hoc test (*P* < 0.05) was performed on the log_2_ transformed data (Calibrated Normalized Relative Quantities CNRQs) using Statistica 12 software (Statsoft Inc.).

## Electronic Supplementary Material

Supplementary Information
