# Supplementary material for: Identification of reference genes for RT-qPCR data normalization in Gammarus fossarum (Crustacea Amphipoda)
Source: Sci Rep. 2018 Oct 15;8:15225. doi: 10.1038/s41598-018-33561-1 (PMC6189083; doi:10.1038/s41598-018-33561-1)
Supplement: Supplementary file 1 — Supplementary Information [file 41598_2018_33561_MOESM1_ESM.docx]

Identification of reference genes for RT-qPCR data normalization in *Gammarus fossarum* (Crustacea Amphipoda)

Kahina Mehennaoui ^1,2^, Sylvain Legay ^1^, Tommaso Serchi ^1^, François Guérold ^2^, Laure Giambérini ^2^, Arno C. Gutleb ^1^, Sébastien Cambier ^1*^

^1^ Environmental Research and Innovation (ERIN) Department, Luxembourg Institute of Science and Technology (LIST), 5, avenue des Hauts-Fourneaux, Esch-sur-Alzette, Luxembourg; [kahina.mehennaoui@list.lu](mailto:kahina.mehennaoui@list.lu) (K.M.); [sylvain.legay@list.lu](mailto:sylvain.legay@list.lu) (S.L.); [tommaso.serchi@list.lu](mailto:tommaso.serchi@list.lu) (T.S.); [arno.gutleb@list.lu](mailto:arno.gutleb@list.lu) (A.C.G.)

^2^ Laboratoire Interdisciplinaire des Environnements Continentaux (LIEC), CNRS UMR 7360, Université de Lorraine – Metz, France ; francois.guerold@univ-lorraine.fr (F.G.) ; [laure.giamberini@univ-lorraine.fr](mailto:laure.giamberini@univ-lorraine.fr) (L.G.)

***** Correspondence: [sebastien.cambier@list.lu](mailto:sebastien.cambier@list.lu) (S.C.); Tel: +352-275-888-5018; fax: +352 275 885

**Table S1:** Identification of *Gammarus fossarum* gene sequences.


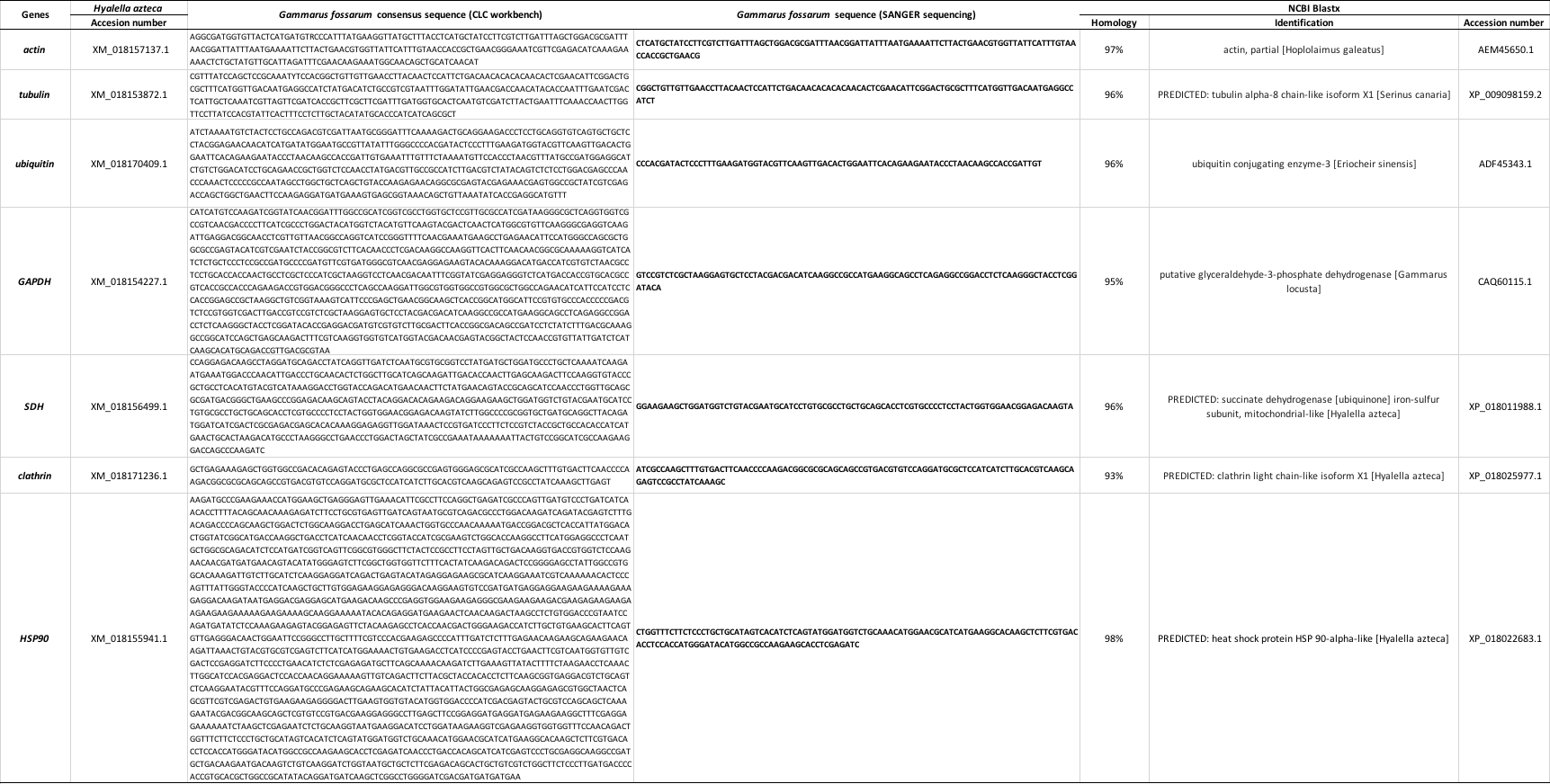


**Table S2:** Cq values (mean ± SD) of the different genes regarding the different exposure conditions.

| **Compound ^a^** | | **Concentrations (µg.L^-1^)^b^** | ***Actin*** | ***Clathrin*** | ***GAPDH*** | ***SDH*** | ***TUB*** | ***UB*** | ***HSP90*** |
| --- | --- | --- | --- | --- | --- | --- | --- | --- | --- |
| **AgNO_3_** | | **0** | 21.60 ± 0.57 | 21.40 ± 0.30 | 18.69 ± 0.52 | 19.99 ± 0.40 | 18.60 ± 0.28 | 24.16 ± 0.34 | 18.73 ± 0.48 |
|  |  | **0,5** | 20.93 ± 0.30 | 21.23 ± 0.04 | 18.93 ± 0.20 | 19.82 ± 0.13 | 18.01 ± 0.22 | 23.86 ± 0.11 | 18.48 ± 0.20 |
| **AgNPs 40 nm** |  | **0** | 21.35 ± 0.21 | 21.12 ± 0.57 | 18.65 ± 0.13 | 20.21 ± 0.08 | 18.39 ± 0.11 | 23.39 ± 0.60 | 19.22 ± 0.10 |
|  | **CIT** | **0,5** | 22.22 ± 0.24 | 21.32 ± 0.03 | 18.52 ± 0.38 | 20.23 ± 0.10 | 17.97 ± 0.21 | 23.85 ± 0.12 | 18.78 ± 0.09 |
|  | **PEG** | **0,5** | 21.67 ± 0.10 | 21.34 ± 0.05 | 18.60 ± 0.21 | 20.31 ± 0.03 | 18.02 ± 0.32 | 23.94 ± 0.12 | 19.03 ± 0.29 |
| **AuNPs 40 nm** |  | **0** | 21.55 ± 0.17 | 21.21 ± 0.13 | 18.78 ± 0.14 | 20.17 ± 0.06 | 18.07 ± 0.30 | 24.01 ± 0.05 | 18.57 ± 0.28 |
|  | **CIT** | **0,5** | 21.45 ± 0.15 | 21.25 ± 0.08 | 18.85 ± 0.18 | 20.07 ± 0.03 | 18.14 ± 0.29 | 24.00 ± 0.11 | 19.16 ± 0.09 |
|  | **PEG** | **0,5** | 21.56 ± 0.15 | 21.26 ± 0.13 | 18.71 ± 0.20 | 19.98 ± 0.08 | 18.69 ± 0.13 | 23.58 ± 0.60 | 19.04 ± 0.17 |

^a^ CIT: citrate stabilized NPs, PEG: polyethylene-glycol coated NPs

^b^ 0: this condition is the control condition for each compound

**Table S3:** Cq values (mean ± SD) of the different genes regarding the calibration curves experiment.

| ***G. fossarum cDNA***  ***concentrations (ng)*** | ***Actin*** | ***Clathrin*** | ***GAPDH*** | ***SDH*** | ***TUB*** | ***UB*** | ***HSP90*** |
| --- | --- | --- | --- | --- | --- | --- | --- |
| **0.04** | <LOD | <LOD | <LOD | <LOD | <LOD | <LOD | <LOD |
| **0.2** | 26.03 ± 0.14 | 25.93 ± 0.02 | 24.40 ± 0.02 | 25.40 ± 0.09 | 23.45 ± 0.06 | 27.19 ± 0.41 | 24.57 ± 0.02 |
| **1** | 23.77 ± 0.09 | 23.49 ± 0.10 | 21.82 ± 0.04 | 23.10 ± 0.07 | 21.16 ± 0.05 | 24.79 ± 0.12 | 21.98 ± 0.08 |
| **5** | 21.59 ± 0.12 | 21.11 ± 0.14 | 19.34 ± 0.01 | 20.61 ± 0.08 | 19.09 ± 0.09 | 22.59 ± 0.18 | 19.50 ± 0.05 |
| **25** | 22.72 ± 0.05 | 18.96 ± 0.03 | 16.95 ± 0.04 | 18.42 ± 0.03 | 18.11 ± 0.17 | 20.78 ± 0.18 | 17.61 ± 0.03 |

<LOD: under limit of detection of ViiA7 qPCR system

**Table S4:** MIQE reporting for the experiment of this study using template (Remans et al., 2014).

| **Sample/Template** | **Experiment** | **Checklist** |
| --- | --- | --- |
| **Source** | Adult *Gammarus fossarum* males that were acclimated to the laboratory conditions | Ѵ |
| **Method of preservation** | Liquid N2 snap freeze, -80°C | Ѵ |
| **Storage time (if appropriate)** | < 6 months | Ѵ |
| **Handling** | Frozen | Ѵ |
| **Extraction method** | QIAGEN RNeasy mini kit | Ѵ |
| **RNA:DNA free** | Check absence of PCR amplification on RNA + Check melting curve | Ѵ |
| **Concentration** | Nanodrop | Ѵ |
| **RNA: integrity** | Bioanalyzer of a small volume of all samples | Ѵ |
| **Inhibition free** | Parallel amplification plots in log-linear phase | Ѵ |
|  |  |  |
| **Assay optimization/validation** | | |
| **Accession number** | See table 2 about gene sequence identifications in m&m | Ѵ |
| **Amplicon details** | See table 3 about primers in m&m and table SXX Supplementary Material | Ѵ |
| **Primer sequence** | See table 3 about primers in m&m | Ѵ |
| **Probe sequence** | n/a |  |
| **In silico** | NCBI Blast “Other (nr etc.)” | Ѵ |
| **Empirical** | 100nM / 60°C annealing | Ѵ |
| **Priming conditions** | Random hexamers | Ѵ |
| **PCR efficiency** | Dilution curve of pooled sample | Ѵ |
| **Linear dynamic range** | 5-fold dilution over 5 points calibration curve | Ѵ |
| **Limits of detection** | n/a |  |
| **Intra assay variation** | n/a |  |
|  |  |  |
| **RT/PCR** | | |
| **Protocols** | See m%m | Ѵ |
| **Reagents** | See m&m | Ѵ |
| **Duplicate RT** | Not used |  |
| **NTC** | Cq & melting curves checked = OK | Ѵ |
| **NAC** | Cq & melting curves checked = OK | Ѵ |
| **Positive control** | n/a |  |
|  |  |  |
| **Data analysis** | | |
| **Specialist software** | QuantStudio™ Real-Time PCR Software, MS Excel | Ѵ |
| **Statistical justification** | 4 biological replicates composed of a pool of4 individuals, one Way ANOVA with Post | Ѵ |
| **Transparent, validated normalisation** | GeNorm analysis and others (see m&m) | Ѵ |

*
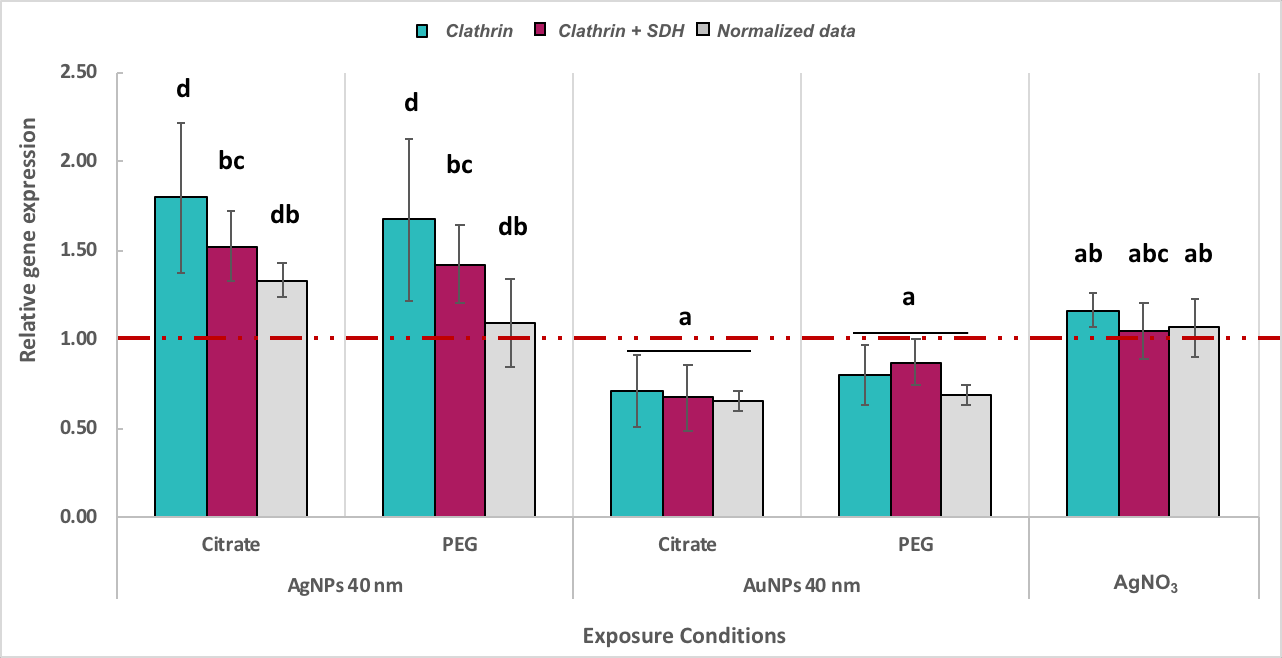
*

**Figure S1:** Representation of the RT-qPCR data including the uncertainty of the accuracy. *HSP90* expression analysis was performed using *Clathrin* (Blue) and *Clathrin+SDH* (dark purple) non-normalized data and with normalized data (grey). Error bars indicate the standard errors of the means (n=4). Different letters (a-d) indicate significant differences at *P* < 0.05. Red line indicates control groups with relative gene expression = 1.

**Figure S2**: Calibration curves of quantified reference and target genes on *G. fossarum*. PCR efficiencies for all tested primers were evaluated using decreasing five-fold dilutions from cDNA pool (from 25 ng to 0.04 ng and no template control).

**Figure S3:** Melting plot curves of tested primers for reference and target genes of *G. fossarum.*
